# An evaluation of the Africa-CORDEX regional climate model's performance in simulating air temperatures and precipitation in the Melka-Wakena catchment, southeast Ethiopia

**DOI:** 10.1016/j.heliyon.2024.e40720

**Published:** 2024-12-05

**Authors:** Tadele Sh Gerasu, Tolera Abdissa Feyissa, Beekan Gurmessa Gudeta, Keneni Demissie, Mulatu Tesfahun

**Affiliations:** a Department of Hydraulic and Water Resource Engineering, Jimma University Institute of Technology, P.O. Box 378, Jimma, Ethiopia; b Department of Water Supply and Environmental Engineering, Jimma University Institute of Technology, P.O. Box 378, Jimma, Ethiopia

**Keywords:** CORDEX-AFR, RCM performance, Melka-Wakena Ethiopia, Precipitation, Temperature, Taylor diagram, Multi-metric weighted ranking, Hybrid approach

## Abstract

Understanding climate science is essential for effective policy development, adaptation, mitigation, and risk management. Given the inherent limitations in climate models, this study evaluates the performance of CORDEX Africa regional climate models to simulate precipitation and temperatures over the Melka-Wakena catchment. To accomplish this, the performance evaluation utilizes techniques such as multi-metric weighted ranking to select top-1 (best individual model), specific multi-model ensembles (top-N ensemble), multi-model ensemble, and average hybrid (top-N ensemble with MME) approaches at various temporal scales. Also, Taylor diagrams and empirical cumulative distribution functions are other useful elements for model comparison visualization. This study finds that, top-3 ensemble (for temporal scale tasmin, monthly pr), top-7 ensemble (monthly and seasonal tasmax), average hybrid (top-1 with MME (daily pr and tasmax), HadGEM2ES_RACMO22T for seasonal and yearly pr), and HadGEM2ES_RCA4 for yearly tasmax) are preferable, with weighted scores of 0.29, 0.44, 0.22, 0.20, 0.35, 0.40, 0.39, 0.33, 0.45, 0.25, and 0.33, respectively. In short, RCA4 and RACMO22T efficiently mimic air temperatures and precipitation in the Melka-Wakena catchment. While ensemble approaches are generally more resilient and possible, single-best-performing model approaches can be quite effective for long-term periods. Finally, these studies have an important implication for improving climate change assessment, adaptation, and mitigation measures.

## 1 Introduction

The effects of climate change are of great concern and are worsening, especially in developing nations [1,2]. The frequency, severity, spatial extent, length, and timing of weather and climate extremes have all changed because of changes in the frequency and intensity [3,4]. Climate science has long faced the challenge of providing precise estimates of climate change at a high spatial resolution [5,6]. High-resolution projections are essential to adapt to climate change and prepare for future changes in extreme events [5,6]. Many climate assessment studies are necessary to understand the effects of climate variability and change on economic sectors such as agriculture and food, water resources, energy, and transportation. Its catastrophic effects, including prolonged droughts, heat waves, and flooding, have been well documented over the last few decades. Also, the provision provides a scientific basis for regional policy and decision support, adaptation issues, mitigation planning, and risk management strategies [[7], [8], [9]].

According to Refs. [8,10,11] of the Intergovernmental Panel on Climate Change, the tremendous global industrial revolution that grew over the past 50 years has coincided with a dramatic rise in the surface temperature of the Earth. The main contributor to global warming and climate change is greenhouse gas (GHG) emissions from anthropogenic activities [4,8]. Also, interconnected hydrological cycles, biodiversity, and ecosystem health [11], climate change may affect various human endeavors [4,12,13].

The earlier research suggests that [7,10,12], global climate models (GCMs) assess and evaluate climate change and its variability in different regions. However, because of the high computational and storage requirements, the GCMs are only possible with a coarse spatial resolution of 100–250 km [12,14,15]. In addition, the GCMs assess the impact of heterogeneity in orographic vegetation and soils on climate systems at a regional level [8,12,14,[16], [17], [18]]. Reliable climate change information at finer spatial scales is required to develop climate change adaptation policies [16,19] To offer regional climate model information at a local scale for historical and future periods, GCMs are dynamically or statistically downscaling [10,16,18,20].

As a result, no single global climate model is accurate, and each model's climate modeling and projections are subject to varying degrees of uncertainty [7,14,18,[20], [21], [22]]. Thus, the multi-model ensemble with consideration of all available climate models (regardless of their performance) is the widely used approach in many published research works [7,14,21,[23], [24], [25], [26]]. This raises doubts about the blind multiple-model ensemble approach's use [21].

Statistical downscaling (data-driven) models are mathematical relationships between local climate variables and large-scale predictors. They apply and require much less computational time than dynamic down-scaling approaches, which are process-driven physical models with high-resolution regional climate models [7,21]. However, regional climate model (RCM) downscaling factors include boundary situations, (GHG) emission situations, configuration, atmosphere ocean Global Climate Model inner variability, regional climate downscaling (RCD) configuration, inner variability, and technique, and region of interest [3,10,20]. The accuracy and performance of each RCM may vary geographically [20,[27], [28], [29]].

According to various studies [6,7,15,20,23,[30], [31], [32], [33]] CORDEX offers a standard for assessing and potentially improving models and a series of experiments that enable us to investigate the impact of various sources of uncertainty. The World Climate Research Program (WCRP) is funding the Coordinated Regional Down Scaling Experiment (CORDEX), which aims to improve the framework for producing regional-scale climate projections within the IPCC AR5 timeline and beyond [9,14,18,29,34]. The ensemble of CORDEX climate projection experiments is forced by either reanalysis or GCM data (model forcing), while RCP (representative concentration pathways) is responsible for radiation forcing scenarios [2].

Unfortunately, Africa and Ethiopia are among the region's most vulnerable to the consequences of climate change [6,13,21,35] However, earlier research conducted in Ethiopia showed the need for a thorough assessment to determine the uncertainty of several climate models [18,34]. These uncertainties are also the result of various theories, applied methods, and initial boundary conditions [2,32] Climate projections from various climate models vary [9,11,22,34], and sources of uncertainty in climate projections vary [7,15,33]. Global climate models continue to face challenging tasks, such as reproducing existing climatic conditions and predicting potential future changes in weather extremes at a regional scale [4].

The effectiveness of the RCM in capturing the traits of weather diversity has received a great deal of attention [15,23,24,33] over the Melka Wakena catchment in southeast Ethiopia, there is no assessment of a model's performance in simulation from AFR-44 or the AFR-44 ensemble. Therefore, this study used AFR-44 model performance evaluation to remedy this flaw and to use a better climate model for impact and adaptation assessment.

However, climate model evaluation for the region of interest is helpful for impact assessment [36]. This study's main aim is to evaluate the performance of the AFR-44 regional climate models across the Melka Wakena region of southeast Ethiopia. This study specifically aims (i) to assess AFR-44 RCM performance in simulating air temperature and precipitation over Melka Wakena, southeast Ethiopia. Second, (ii) to choose preferable models for usage in upcoming simulations for generating future climate variable projection scenarios. How much better is the simulation from AFR-44 or the AFR-44 ensemble at projecting the precipitation and temperature patterns in southeast Ethiopia? Which RCM models outperform others?

In addition, a study by Ref. [37] analyzed the impact of the climate on stream flow using a single climate model, performing no prior analysis of AFR-44 RCM performance over the area of interest, which is a gap. As yet [7,18,22,36] there is uncertainty in climate models; the projected climate models or simulations are either underestimating or overestimating climate conditions, and ensemble or individual models before pre-evaluation climate models do not imply agreement with reference data. Therefore, rather than using single climate models or ensemble multiple models [18,36,37], it is imperative to assess the performance of the AFR-44 RCM as ensemble-specific multi-models (top-N models ensemble) [29,38] and hybrid approach. Choosing a few GCM-RCMs to reflect the ensemble is therefore a general problem to be addressed. It is, of course, always advisable to choose the possible GCM-RCM; yet, numerous studies have demonstrated that the possible model varies depending on the parameter or parameters being studied as well as regionally [39].

Previous studies [6,13,14,21,26,29,32,38] have employed spatiotemporal patterns and different climatic conditions and temporal scales over areas of interest to assess the performance of climate models. The empirical Cumulative Distribution Function (empirical CDF), multi-criteria decision method [13,17,38], and statistical [36,38] analyses help select the best climate models for the climate condition. It gets challenging when multiple models run simultaneously with varying parameters and conditions [12].

This look evaluates AFR-44 RCMs over the Melka-Wakena catchment in Southeast Ethiopia using an empirical CDF, Taylor diagrams, a statistical metrics test, and a Taylor skill score. The Taylor diagram helps visualize the results of climate models in a single plot [24,35]. R software for Taylor diagram, which comes with R packages such as chronological objects, which can handle dates and times (Chron), Trellis Graphics for R (lattice), a variety of plotting functions package, and other techniques to create beautiful data visualizations.

A Taylor approach effectively draws a graphic representation of the observed and modeled data [28]. An azimuth angle in the Taylor diagram shows the correlation coefficient between the modeled and the observed data [16,28]. A radial distance from the origin shows the normalized standard deviation of the simulation compared to that of the observation, according to research by Refs. [24,32,40]. Taylor's diagram represents these elements using polar coordinates.

## 2 Material and methods

### 2.1 Area of interest (AOI)

**Fig. 1:**
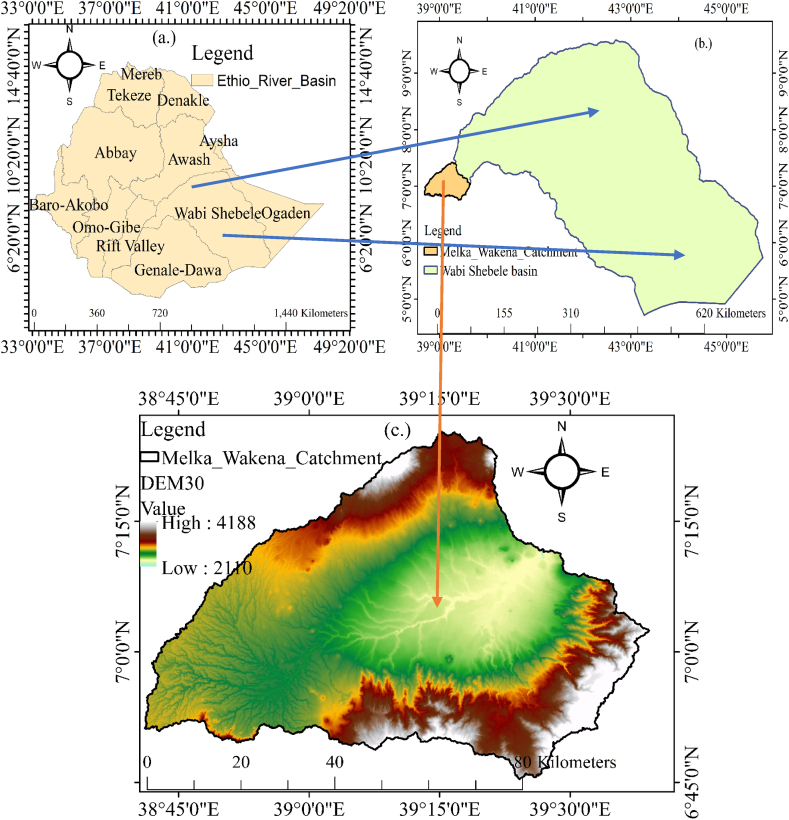
Study area map: a. Ethio-Basin b. Wabe-Shabele Basin, and c. Melka-Wakena Catchment.

According to Fig. 1, the research study was conducted over the Melka Wakena watershed at the source of the Upper Wabi Shebelle river basin in Ethiopia's Oromia Region. Its coordinates are 6.5–7.5°N latitude and 38.15–39.7°E longitude.

The watershed, which has a surface area of 4380-km square, is one of the main tributaries of the Upper Wabi Shebelle River basin. Adaba, Dodola, Gedebi-Assessa, Kofale, Kore, Kokkosa, and Lemu Bilbilo (Meraro) are some of the seven districts. The watershed's elevation varies from 2243 to 4178 m above sea level.

### 2.2 Data preparation and analysis

#### 2.2.1 Source of data

This study examines the performance of the Africa-CORDEX Regional Climate Models using a variety of datasets. The Ethiopian Meteorological Agency (EMA) provided the major data sources, which included a digital elevation model (DEM) and observed meteorological data (precipitation, maximum and minimum temperatures, humidity, and wind speed) from 1991 to 2005. The Earth System Grid Federation (ESGF) provides climate model data, with a focus on historical and future projections from the CORDEX project. The ESGF portal (https://esgf-node.llnl.gov) allowed users access to historical precipitation and temperature data important to the study areas.

#### 2.2.2 Data preparation and extraction

Preprocessing the climate model outputs into formats appropriate for study was part of the data preparation process. To get ready for the model evaluation, the data was reorganized into daily, monthly, seasonal, and annual formats. As a result, extraction processes were carried out and the climate model data files that may be downloaded are in the NetCDF4 file format. Utilizing the NetCDF4 package for data extraction, evaluation metrics computation, and data visualization (Taylor diagram, scatter plot, and empirical cumulative distribution function), the R and Jupiter programming languages were used for this study.

**Fig. 2:**
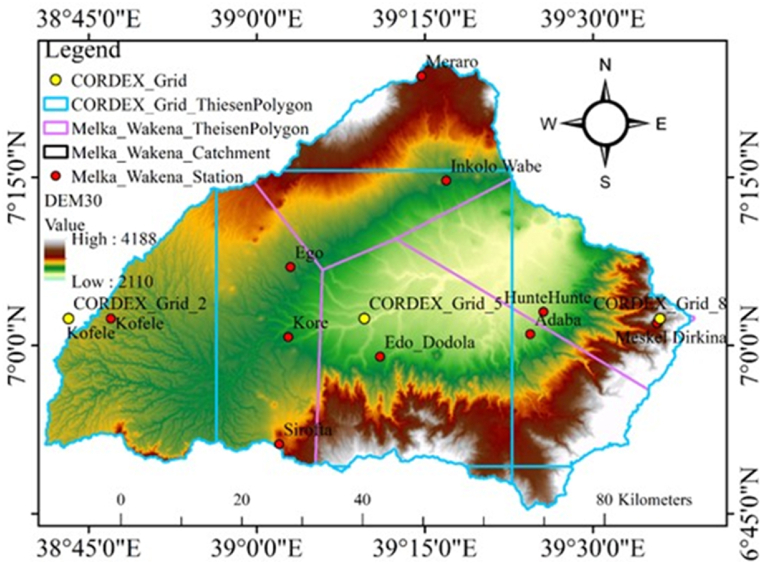
Melka Wakena Catchment's Theissen polygon and the ETH CORDEX Grid.

For this investigation, CORDEX AFR44 grids were used to determine grid points inside the study area's extraction border. A Thiessen polygon and a mask delineating the watershed border were created using Geographic Information System (GIS) software. These CORDEX grid points were critical in obtaining regional climate model data for the Melka Wakena catchment. The user entered CORDEX grid points for the Melka Wakena Catchment, also known as the Melka Wakena CORDEX Grid. CORDEX Grid _2 is situated at latitude 7.04 and longitude 38.72, CORDEX Grid _5 at latitude 7.04 and longitude 39.16, and CORDEX Grid _8 at latitude 7.04 and longitude 39.60, as shown in Fig. 2.

#### 2.2.3 Bias correction and Lapse Rate Adjustment

The primary aim of this work was to compare model outputs directly to observed data, hence bias adjustment was not used. Prior to the orographic effect, model outputs may persistently overestimate or underestimate station-level temperature and precipitation, resulting in model biased [7]. Meteorological stations are also best located in valleys or at lower altitudes than the average grid point elevation. Initially, Lapse Rate Adjustment was used to correct for variations between model and station levels. This adjustment accounts for the reduction in air temperature that occurs as altitude increases. However, considering the low elevation difference in the Melka Wakena watershed, the grid elevation effect appeared insignificant for this study.

#### 2.2.4 Fill in the missing data

One of the most important steps of data analysis is assuring data quality. The efficiency with which data is processed influences the work's final outcome. As a result, missing data in datasets is a concern, and several strategies are being used to fill in the gaps. The percentage of missing reference station data ranged from 11% to 33 %, 13%–21 %, and 14%–18 % for precipitation, maximum temperature, and minimum temperature for the entire period. The Multiple Imputation Chainage Equation (MICE) package from the R statistical computer environment was critical to this work. MICE is a statistical strategy for handling missing data. Since, to handle missing datasets. This method generates multiple imputed datasets, capturing the uncertainty caused by missing values. The technique is done multiple times, or "chained," until the imputations are stable. The MICE package in R was used to fill in missing data, making the statistical analysis more robust.

### 2.3 AFR-44 regional climate model

Table 1 lists the nine global climate models (GCMs) and three regional climate models (RCMs) used in this study. Model outputs were compared to observed data from 1991 to 2005. The study emphasized on historical periods without using bias corrections because the primary goal was to compare model simulations to actual observations. Bias corrections are required when utilizing climate model simulation data to assess impacts.

**Table 1:** GCM-RCM subset.

GCM	RCM	RCMs Reference
CNRM-CERFACS-CNRM-CM5	RCA4	[41]
CSIRO-QCE-CSIRO-Mk3-6-0	RCA4	[41]
ICHEC-EC-EARTH	HIMR_HIRHAM5	[42]
ICHEC-EC-EARTH	RACMO22T	[43]
ICHEC-EC-EARTH	RACMO22T(r12v1)	[43]
ICHEC-EC-EARTH	RCA4	[41]
ICHEC-EC-EARTH	RCA4(r3v1)	[41]
IPSL-IPSL-CM5A-MR	RCA4	[41]
MIROC-MIROC5	RCA4	[41]
MOHC-HadGEM2-ES	RAC4	[41]
MOHC-HadGEM2-ES	RACMO22T	[43]
MPI-M-MPI-ESM-LR	RCA4	[41]
MPI-M-MPI-ESM-LR	RCA4(r3v1)	[41]
NCC-NorESM1-M	RCA4	[41]
NOAA-GFDL-GFDL-ESM2M	RCA4	[41]

CORDEX data usage license under Creative Commons Attribution 4.0 International (CC BY 4.0).

**Fig. 3:**
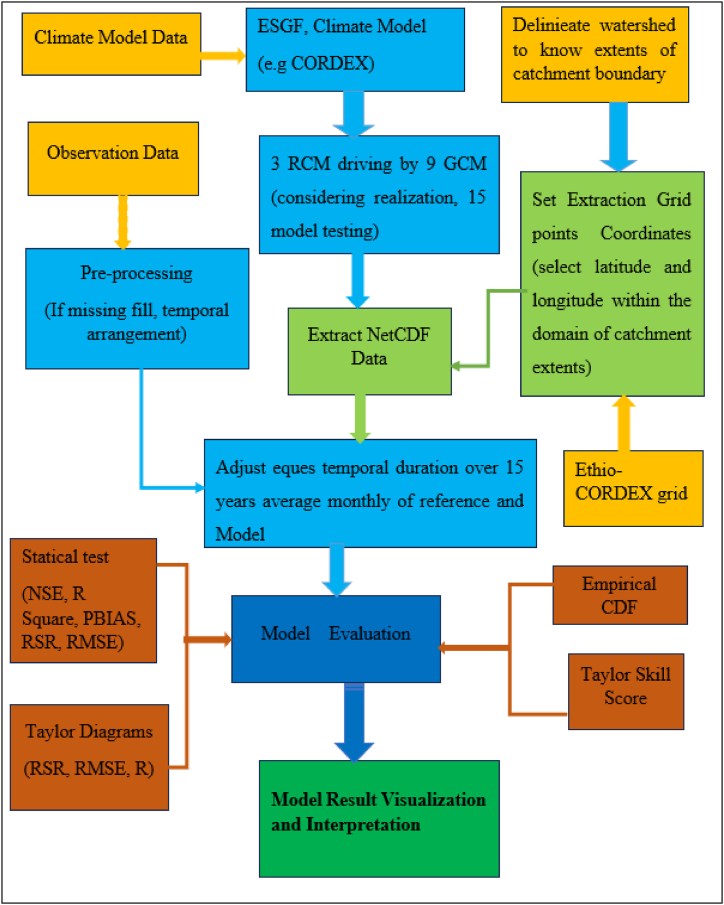
Schematic diagrams represent the performance evaluation of AFR-44 RCMs.

Fig. 3 illustrates the AFR-44 regional climate model performance evaluation procedure. The following established selection criteria in the ESGF portal data: project and domain. CORDEX AFR-44), variable (pr, tasmax, tasmin), experiment (historical, RCP4.5, & RCP8.5), daily period, ensemble r1i1p1, and downscaling realization are all presented in Table 1.

### 2.4 Evaluation performance indicators

#### 2.4.1 Multi-metric weighted ranking system

The multi-metric weighted ranking approach provides a possible mechanism for assessing model performance by combining multiple measures into a single score. This approach addresses variations in individual metrics while ensuring an integrated assessment across many evaluation criteria. By assigning appropriate weights to these metrics, the system ensures that the model's performance is evaluated comprehensively, reflecting its accuracy, fit, and reliability across different dimensions.

$$\text{Weighted}\;\text{Score}=\sum_{i=1}^{n}\left(\text{Metric}\;i*\text{Weight}\;i\right)\;$$

Where: Metric i is the value of the ii-th metric, and Weight i is its associated weight. A number of performance indicators were used to assess model evaluation, including Modified Nash-Sutcliffe Efficiency (NSE), Root Mean Square Error (RMSE), Coefficient of Determination (R2), and Percent of Bias (PBIAS). These criteria were chosen to give a thorough evaluation of model accuracy. Lower RMSE, PBIAS, and RSR values suggest better model performance, but higher NSE, TSS, and R^2^ values imply stronger agreement between model outputs and observed data. Each metric was determined using established formulas, as described in Equation (1) for NSE determination, Equation (2) adjust RMSE, Equation (3) calculates Coefficient of determination, and Equation (4) represent Percent of Bias (PBIAS) provided.

1. Modified Nash-Sutcliffe efficiency (NSE):

NSE values occur over a wider range (from −∞ to positive 1) than the expected typical relative error measure with standard values of zero and one characterizing imperfect and perfect model, respectively.

Equation 1 $$\text{NSE}=1-\left[\;\frac{\sum_{n=1}^{i=N}\;{\left(O_{i}-m_{i}\right)}^{2}\;}{\sum_{i=1}^{\mathrm{ii}=N}{\;\left(0_{i}-\bar{O}\right)}^{2}\;}\right]$$

2. Root means square error (RMSE):

RMSE is the absolute error of climate models when simulating climate variables and measures the difference between RCM results and actual observational climatology. RMSE has the same unit as the observed variable, making its interpretation relatively simple. An RMSE value close to zero indicates favorable performance.

Equation 2 $$\text{RMSE}=\sqrt{\frac{1}{N}\frac{{\sum_{i-1}^{i-N}\left(O_{i}\right)-(m_{i)}}^{2}}{\sum_{i-1}^{I-N}\left(O_{i}-\bar{O}\right)}})$$

3. Coefficient of determination (R^2^):

The correlation coefficient is used in this study and its mathematical formulation is shown below. Its value is between 1 and −1. A positive indicates a strong positive relationship, while a negative indicates a strong negative relationship, and zero indicates a weak or no relationship.

Equation 3 $$R^{2}=\frac{\frac{1}{N}\sum_{n=1}^{N}\left(O_{i}-\bar{O}\right)\left(m_{n}-\bar{m}\right)}{\;\sqrt{\left\{\sum_{n=1}^{N}{\left(O_{i}-\bar{O}\right)}^{2}\right\}}\;\sqrt{\left\{\sum_{n=1}^{N}{\left(m_{n}-\bar{m}\right)}^{2}\right\}}}$$

4. Percent of Bias (PBIAS)

The absolute percentage biases between the regional climate model findings and the observed climate were calculated for precipitation and temperature. The absolute percentage bias is the systematic error between the RCM and the actual climatology of the ground station. Positive numbers imply that climate models are overestimated, whereas negative values indicate that they are underestimated. The values closest to 0 represent the smallest systematic difference and ensures the climate models' best estimates.

Equation 4 $$\text{PBIAS}=100\;\left[\;\frac{\sum \left(\mathrm{sim}-\mathrm{obs}\;\right)\;}{\sum \left(\mathrm{obs}\right)\;}\right]$$

Where: RMSE = centered root mean squared error*,* $\sigma_{O}$ = standard deviation of observed values, $\sigma_{S}$ = standard deviation of simulated values*,* $\bar{m}$ = mean of the model simulated time series*,* $\bar{O}$ = mean of the observed time series*,* $O_{i}$ = Observed time series, $m_{n}$ = simulated time series, and N is the number of data points.

5. Additional metrics used in this system include:

Mean Squared Error (MSE): Calculates the average of the squared errors, reflecting overall prediction accuracy and punishing greater errors more than smaller ones. Mean Absolute Error (MAE): This is the average size of errors in forecasts, showing the average deviation without squaring errors. Kling-Gupta Efficiency (KGE) combines bias (αα), variability (ββ), and correlation (r) into a single statistic to assess overall model performance.

In general, the lower the error measures (bias and RMSE), the better the model's performance. The correlation coefficient might range from negative one (perfect negative correlation) to one (perfect positive correlation) between the RCMs and the observed meteorological variables. In many circumstances, there is no single metrics that consistently identifies the possible RCM. Thus, BIAS, RMSE, and R2 are combined. It is conceivable to have an acceptable R2 variation from one, but no acceptable common value for BIAS or RMSE. In this regard, there is no acceptable limit, except that the lower the BIAS and RMSE, the greater the accuracy.

#### 2.4.2 Evaluation approach

Researchers have evaluated the models using diverse ways from the literature, but there are no acknowledged standard approaches for a single metric performance indicator [40,44,45]. However, this work proposes an evaluation strategy that includes a systematic evaluation of several modeling methodologies in order to select the method for feasible predictions. This method comprises of a few main strategies:

The **top-1 approach** seeks to select the single best-performing model based on the highest weighted score from several evaluation metrics. This strategy emphasizes the model that performs the best across all metrics. Furthermore, the top-N ensemble (specific multi-model ensemble ((SMME)) approach use average estimates among the top N models, where N might be 3, 5, or 7. It seeks to increase predictability by combining the results of top-ranked models. The use of numerous models allows for the identification of a greater range of variances while also reducing individual model bias.

The **multi-model ensemble (MME** technique brings together projections from all available models to create a comprehensive ensemble forecast. This strategy attempts to enhance reliability by using the different outputs of all models, resulting in a more robust prediction that incorporates the strengths of each unique model.

**Hybrid** approaches improve performance by combining forecasts from chosen top-N model ensembles with the MME. These methods include top-1 with MME, top-3 ensemble with MME, top-5 ensemble with MME, and top-7 ensemble with MME. Each hybrid technique averages the predictions of a given number of top models with those of the MME. This integration enables balancing individual model projections with the broader ensemble forecast, resulting in a more nuanced and perhaps more accurate prediction.

The multi-metric weighted ranking system guides the **selection of the best approach** by evaluating the effectiveness of each approach using an extensive evaluation of several performance indicators. This study ensures that the chosen technique gives a comprehensive and balanced examination, making it easier to choose the most effective model or ensemble method for making accurate predictions.

#### 2.4.3 Empirical **cumulative distribution function (empirical CDF)**

The empirical Cumulative Distribution Function (empirical CDF) was used to describe the distribution of observed and modeled datasets [34,37]. The cumulative distribution function of this function increases by 1/n, with a step function at each of the n data points. The cumulative distribution function value is determined by the fraction of observation datasets that are less than or equal to a given measured variable value. The empirical CDF plots provide a clear visual depiction of how well the models reflect observed climate variability, allowing for comprehensive comparisons of various models.

#### 2.4.4 Taylor Diagrams and Taylor skill score (TSS)

Taylor diagrams were employed to visually summarize the performance of each model in terms of correlation, RMSE, and standard deviation [40]. The relationship was plotted on a two-dimensional Taylor diagram using R software. The Taylor Skill Score (TSS) was calculated to provide a comprehensive indicator of model performance [40]. Models that appear closer to the reference point on the Taylor diagram and have higher TSS values are considered to have better agreement with the observed data.

Equation (5) through 8 are used to calculate an evaluation metrics. Equation (5) defines the RSR, Equation (6) calculates the CRMSE, Equation (7) determine correlation coefficients, and Equation (8) ensures about Taylor skill score:

Equation 5 $$1.\;\text{RS}R=\frac{\text{RMSE}}{\sigma_{O}}$$

2. CRMSE

Equation 6 $$\sqrt{\left\{\frac{1}{N}\sum_{i=1}^{N}\;{\left[\left(O_{i}\;\left(\bar{O}\right)-\left(m_{i}\right)\;\bar{m}\right)\right]}^{2}\right\}}$$

3. Correlation Coefficients are defined as:

Equation 7 $$R=\frac{\frac{1}{N}\sum_{i=1}^{N}\left(O_{i}-\bar{O}\right)\left(i-\bar{m}\right)}{\sigma_{O}\;\sigma_{m}}$$

4. Taylor skill score

Equation 8 $$\text{TS}=\frac{4\;{\left(1+R\right)}^{4}}{{\left(\frac{\sigma m}{\sigma_{o}}+\frac{\sigma_{o}}{\sigma_{m}}\right)}^{2}{\left(1+R_{O}\right)}^{2}}$$

Where the maximum correlation coefficient of all models is denoted by R0 (set to 1), and R stands for the correlation coefficient. σm and σ0 represent the standard deviation of the models and the observed data, respectively. A perfect simulation is shown when TS is close to 1, as long as R approaches R0 and σm approaches σ0. Also, Fig. 3 illustrates the performance evaluation of AFR44 RCMs through schematic diagrams.

## 3 Results

### 3.1 AFR-44 RCMs performance for precipitation and temperature variables

This study incorporates multi-metrics performance indicators such as MAE, RMSE, PBIAS, RSR, Correlation, R2, NSE, TSS, KGE, and MSE to identify an appropriate regional climate model for the area of interest. The model performs better for each metrics values, as the model data matches the observed data. The negative value of performance shows the direction of model estimation (underestimation) from the reference data.

However, using single metrics, there is no consistency among all performance indicators over a single model performance evaluation. Prior to this, in order to make metrics consistency, multiple metrics weighted was helpful to ranking and generalize model performance. Thus, the models are ranked to top-n model using weighted scores of multiple metrics. This study desired evaluation approaches such as: top-1 performing model (top-1PM: best individual model), multi-model ensemble (MME), specific multi-model ensemble (SMME this can be prediction from top-N ensemble), and Average hybrid (AH) (top-N ensemble with MME) approaches in order to select suitable paired GCM-RCM for region of study.

For clarification, top-1 performing model is a best model individually performing that designated as top-1PM uses model raw values. MME considers prediction of the average values of all the models. SMME is based on evaluation metrics of the multi-metric weighted ranking technique employed to select specific models (in case of this study, ensemble for each top-N: 3, 5, and 7) and taking the average prediction values of each top-3, top-5, and top-7. Therefore, SMME ensemble formation are considering a particular model ensemble following ranking top-N model based on weighted performance indicators. Hybrid approaches follow the prediction average combination of MME with top-1, ensemble of each top-3, top-5, and top-7. The selection of the model and approach is based on ranking model using multi-metric weighted techniques for each frequency.

However, some models predict underestimation or overestimation that's seen from a single climate model point of view, and which may be happening due to model projection skill due to spatiotemporal uncertainty. Yet these models were chosen to be in the SMME (prediction ensemble from each top-N ensemble), MME, and hybrid approaches because of their suitability for the purpose of ensemble creation.

#### 3.1.1 Minimum temperature

Forecasts of the minimum temperature over a variety of time periods are consistently produced by the top-3 ensemble technique. The weighted scores of 0.29 for daily, 0.25 for monthly, and 0.22 for seasonal estimates show that combining the top three model predictions consistently represents the lowest temperatures. The top-3 ensemble approach has the highest ranking, even for estimations of the annual lowest temperature, with a score of 0.20. This approach continues to be the most reliable throughout a variety of time scales, even with the possibility of future developments in the collection of long-term trends. This consistency shows that ensemble methods are effective in forecasting minimum temperatures throughout a variety of time periods. The ensemble forecast from EARTH_RCA4, CNRMCM5_RCA4, and HadGEM2ES_RACMO22T is used by the SMME of top three ensemble, with potential weighted performance shown in Table 2.

**Table 2:** Minimum temperature weighted performance indicators.

Approach	Multi-metrics weighted score			
	Daily	Monthly	Seasonal	Yearly
Top-1 (HadGEM2ES_RACMO22T)	0.54	0.55	0.49	0.51
Top-3 Ensemble	**0.29**^**(**^**∗**^**)**^	**0.25**^**(**^**∗**^**)**^	**0.22**^**(**^**∗**^**)**^	**0.20**^**(**^**∗**^**)**^
Top-5 Ensemble	0.40	0.44	0.42	0.43
Top-7 Ensemble	0.33	0.42	0.42	0.60
Multi-Model Ensemble	0.63	0.65	0.64	0.68
AH (Top-3 Ensemble with MME)	0.36	0.40	0.40	0.42
AH (Top-5 Ensemble with MME)	0.48	0.54	0.53	0.59
AH (Top-7 Ensemble with MME)	0.46	0.53	0.54	0.65
AH (Top-1 with MME)	0.35	0.30	0.32	0.39

**(∗) indicate the best approach for respected variable and frequency**.

#### 3.1.2 Maximum temperature

Achieving a weighted score of 0.33, the average hybrid (Top-1 + MME) technique appears to be the most successful daily approach for predicting maximum temperature. In order to successfully balance daily temperature changes, this strategy combines the best individual model with a multi-model ensemble. Using forecasts from seven models, the top-7 ensemble approach (score of 0.35) more effectively represents the subtleties of monthly maximum temperatures at the monthly scale. HadGEM2ES_RAC4, EARTH_RACMO22T, MPIESMLR_RCA4, HadGEM2ES_RACMO22T, CSIROMk360_RCA4, NorESM1M_RCA4, and MIROC5_RCA4 are the best seven ensemble members in this instance.

Seasonally, the top-7 ensemble method continues to be effective with a score of 0.40, thereby continuing this pattern. This suggests that one important benefit of using many models is that seasonal temperature changes can be captured more effectively. The "top-1 (HadGEM2ES_RCA4)" model performs excellently for yearly maximum temperature predictions, scoring 0.33, indicating its applicability in long-term temperature forecasting. Consistent performance over various time scales implies that the best individual models are dependable for long-term forecasts, but ensemble methods are better for shorter-term projections. The preferred best approach weighted scores are identified by star icon in Table 3.

**Table 3:** Maximum temperature weighted performance indicators.

Approach	Multi-metrics weighted score			
	Daily	Monthly	Seasonal	Yearly
Top-1 (HadGEM2ES_RCA4)	0.55	0.55	0.547	**0.35**^**(**^**∗**^**)**^
Top-3 Ensemble	0.39	0.429	0.535	0.65
Top-5 Ensemble	0.35	0.369	0.457	0.378
Top-7 Ensemble	0.34	**0.347**^**(**^**∗**^**)**^	**0.40**^**(**^**∗**^**)**^	0.41
Multi-Model Ensemble	0.57	0.63	0.71	0.74
AH (Top-3 Ensemble with MME)	0.38	0.445	0.588	0.71
AH (Top-5 Ensemble with MME)	0.39	0.446	0.561	0.56
AH (Top-7 Ensemble with MME)	0.44	0.47	0.547	0.57
AH (Top-1 with MME)	**0.33**^**(**^**∗**^**)**^	0.35	0.42	0.58

**(∗) indicate the best approach for respected variable and frequency**.

#### 3.1.3 Precipitation

Precipitation RCMs show distinct variances in performance over various time scales. With a weighted score of 0.39, the average hybrid (top-1 with MME) approach is shown to be the most successful for daily precipitation. The daily variability is well captured by this method, which combines predictions from a multi-model ensemble with the best-performing model (HadGEM2ES_RACMO22T). The top-3 ensemble strategy exhibits better robustness when we switch to the monthly scale, with a weighted score of 0.44. The monthly precipitation patterns are more comprehensively represented by this method, which incorporates projections from the top three models (HadGEM2ES_RACMO22T, HadGEM2ES_RAC4, and IPSLCM5AMR_RCA4).

Seasonally, the top-1 (HadGEM2ES_RACMO22T) model performs exceptionally well, scoring 0.45, indicating that seasonal changes can be sufficiently captured by a single high-performing model. Comparably, the same top-1 (HadGEM2ES_RACMO22T) model leads once more for yearly precipitation, demonstrating its superiority in long-term forecasting with a score of 0.25. Taken together, these findings suggest that single-model approaches can be quite effective for longer times, while ensemble methods are especially good for shorter time scales. The preferred best approach weighted scores are identified by star icon in Table 4.

**Table 4:** Precipitation weighted performance indicator.

Approach	Multi-metrics weighted score			
	Daily	Monthly	Seasonal	Yearly
Top-1 (HadGEM2ES_RACMO22T)	0.45	0.45	**0.45(∗)**	**0.25(∗)**
Top-3 Ensemble	0.40	**0.442(∗)**	0.464	0.39
Top-5 Ensemble	0.46	0.47	0.465	0.47
Top-7 Ensemble	0.52	0.51	0.501	0.53
Multi-Model Ensemble	0.62	0.61	0.58	0.75
AH (Top-3 Ensemble with MME)	0.47	0.50	0.486	0.61
AH (Top-5 Ensemble with MME)	0.52	0.52	0.502	0.63
AH (Top-7 Ensemble with MME)	0.55	0.56	0.53	0.66
AH (Top-1 with MME)	**0.39**^**(**^**∗**^**)**^	0.449	0.481	0.54

(∗) indicate the preferable approach for respected variable and frequency.

#### 3.1.4 Scatter plot best performing approach representation

**Fig. 4:**
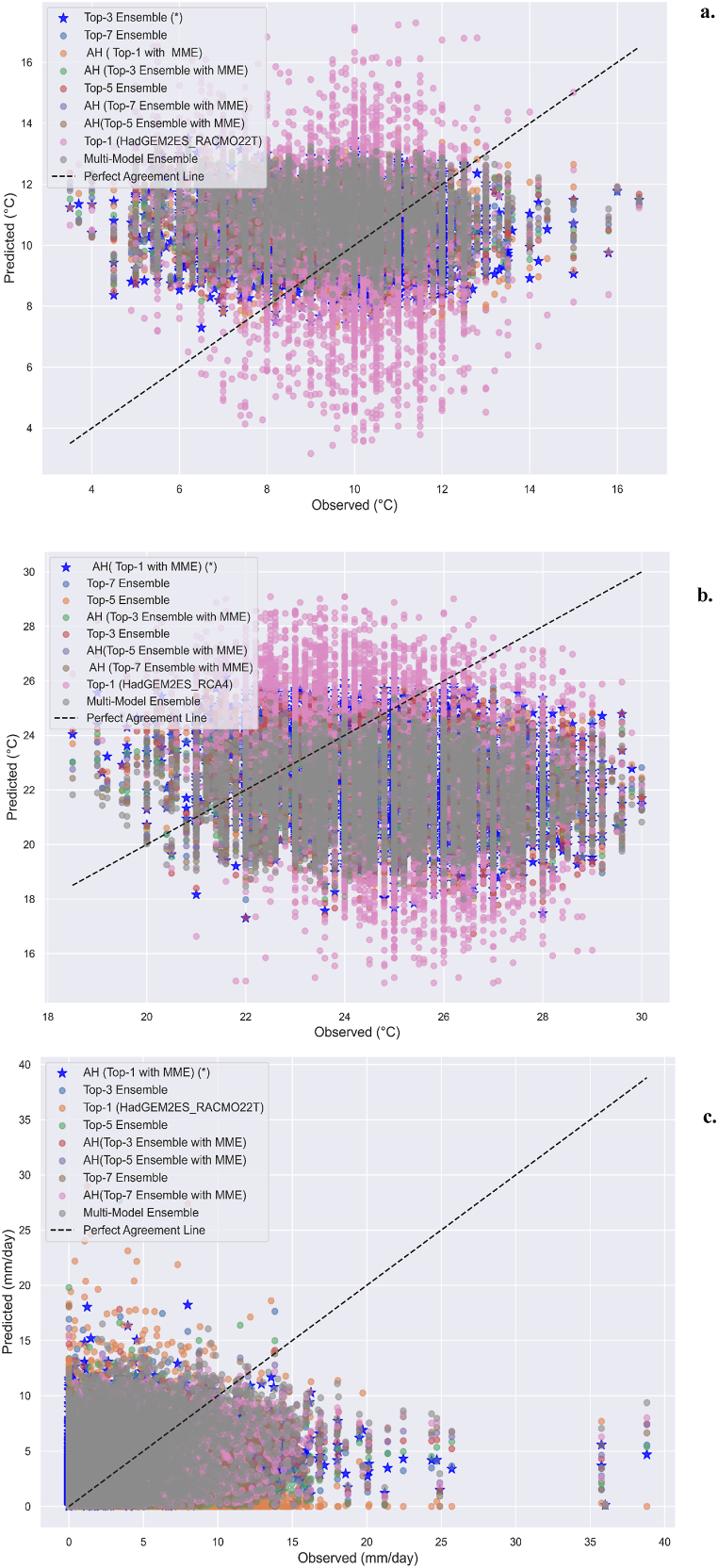
Scatter plotting of daily (1991–2005): a. minimum temperature, b. maximum temperature, c. precipitation.

In Fig. 4(a–c) scatter plot depicts the alignment of observed data with model outputs, highlighting the effectiveness of the best-performing approaches for simulating climate variables in the Melka Wakena catchment. For clarity, each outstanding approach is represented by a star icon. The “AH (Top-1 with MME)” precipitation approach has a significant correlation with daily values recorded, validating its accuracy in short-term forecasts. The “AH (Top-1 with MME)” performs remarkably well in daily maximum temperature predictions, effectively capturing temperature variability. The top-3 ensemble technique governs the distribution of minimum temperatures across all time scales, demonstrating its effectiveness in predicting lower temperature extremes. While, scatter plots for monthly, seasonal, and yearly frequencies of maximum temperature and precipitation are not presented, but similar visualizations confirm that respected approaches consistently perform well across different temporal scales and variables.

### 3.2 Empirical cumulative distribution function

The key statistics (mean, standard deviation, RMSE, maximum, and minimum) of the reference-historical simulated series and for the weather stations in south east Ethiopia for the period daily, monthly, seasonal, and yearly spanning 1991–2005. The climatology feature for the area of interest was computed and used only for the basic analysis of the statical and Taylor diagram plots.

The empirical CDF graphically represents a dataset's feature distribution. The percentage of observations that fall below a certain value is shown. An empirical CDF can be used to plot a data characteristic from lowest to highest. The slope of the empirical CDF provides essential information about the data distribution and variability. For example, a steeper slope indicates less variability in the data (when comparing values), whereas a shorter slope shows greater relative dispersion. The feature datasets, for example, map empirical CDF to insight reference datasets for the top-1 performing model, multi-model ensemble (MME), specialized multi-model ensemble (SMME, which can be forecast from the top-N ensemble), and average hybrid approaches.

**Fig. 5:**
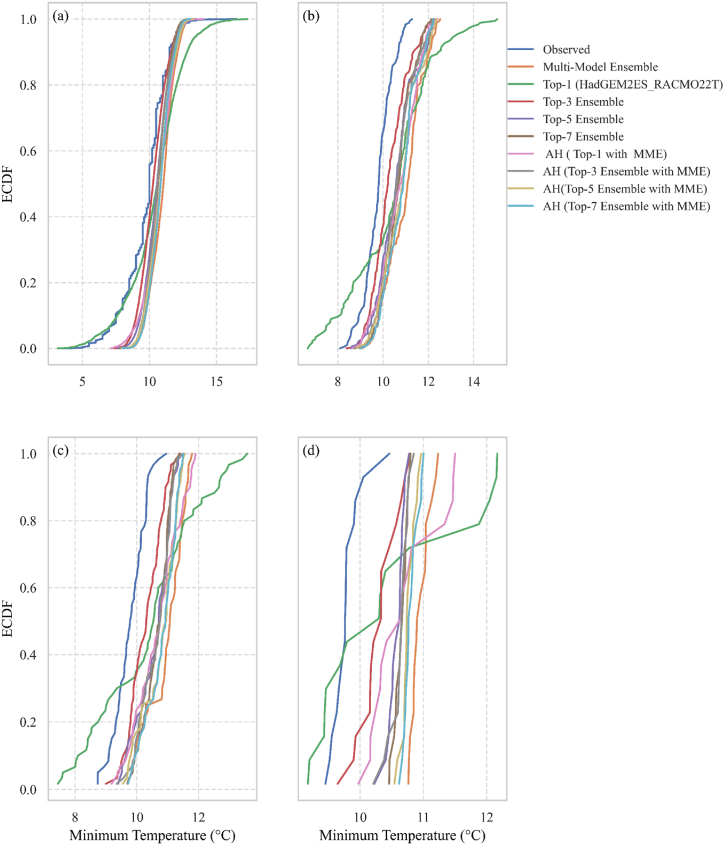
Empirical CDF of Minimum Temperature over 1991–2005: a. Daily b. Monthly c. Seasonal d. Yearly.

Fig. 5(a–d) depicts an empirical CDF analysis of the minimum temperature, demonstrating that the top-3 ensemble technique consistently provides the best match across all temporal scales. This method is remarkably consistent with observed data, whether analyzing daily, monthly, seasonal, or yearly temperature patterns. The ensemble's ability to accurately capture both short-term swings and long-term trends proves its endurance in predicting the minimum temperature throughout many time periods, making it a useful tool for simulating temperature extremes and overall climatological behavior.

**Fig. 6:**
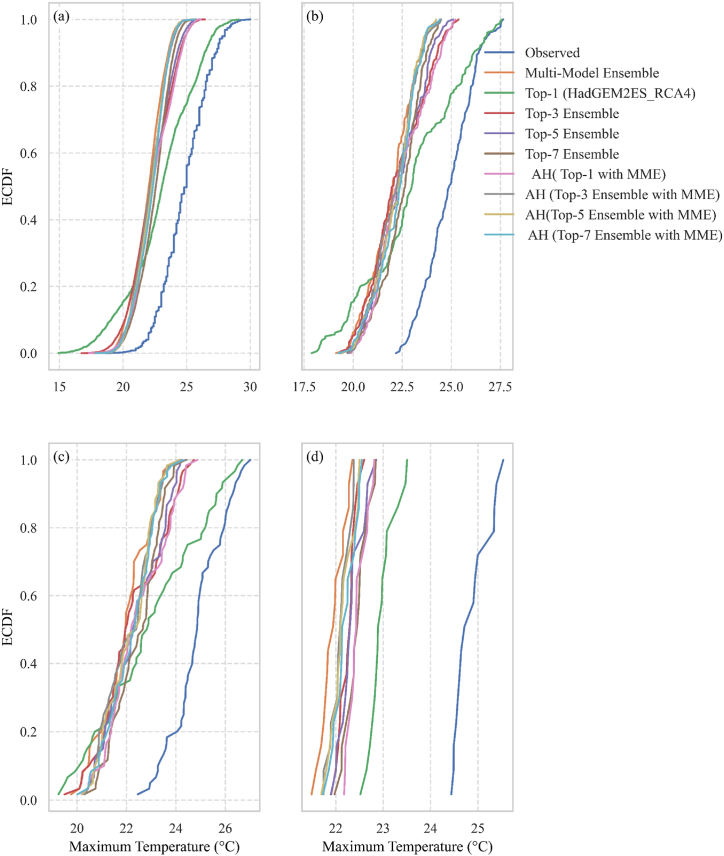
Empirical CDF of Maximum Temperature over 1991–2005: a. Daily, b. Monthly, c. Seasonal, and d. Yearly.

The empirical CDF plot in Fig. 6(a–d) shows that the model's prediction for the highest temperature is consistently lower than the reference data. The empirical CDF analysis for maximum temperature reveals that the AH (Top-1 with MME) strategy corresponds the most closely to observed data on a daily basis, effectively capturing temperature extremes. The top-7 ensemble has better agreement at the monthly and seasonal temporal scales, showing that multi-model aggregation enhances performance. On a yearly scale, top-1 (HadGEM2ES_RACMO22T) provides the closest match, indicating its ability to replicate long-term temperature patterns.

**Fig. 7:**
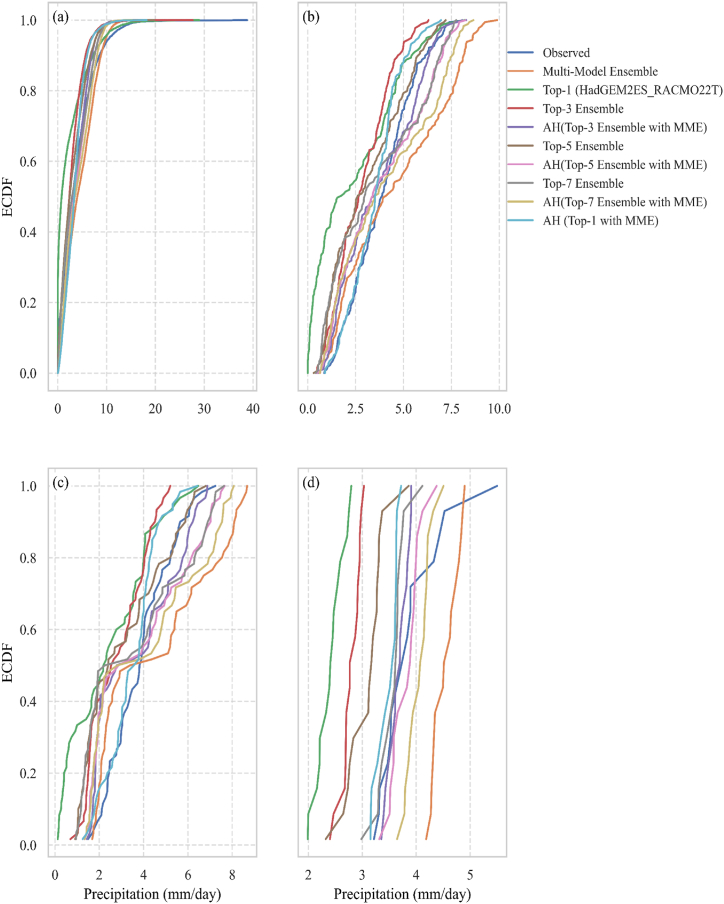
Empirical CDF of Precipitation over 1991–2005: a. Daily, b. Monthly, c. Seasonal, and d. Yearly.

The empirical CDF analysis in Fig. 7(a–d) suggests that different precipitation forecasting approaches function effectively at various temporal scales. For short-term predictions, the AH (Top-1 with MME) technique is particularly reliable since it closely matches observed daily precipitation and adequately accounts for daily variability. The top-3 ensemble approach demonstrates its strength in simulating by fitting well with observed data for monthly precipitation. The top-1 (HadGEM2ES_RACMO22T) approach consistently matches observed patterns on seasonal and yearly timescales, indicating its ability to accurately describe seasonal and annual total fluctuations.

### 3.3 Taylor Diagram

**Fig. 8:**
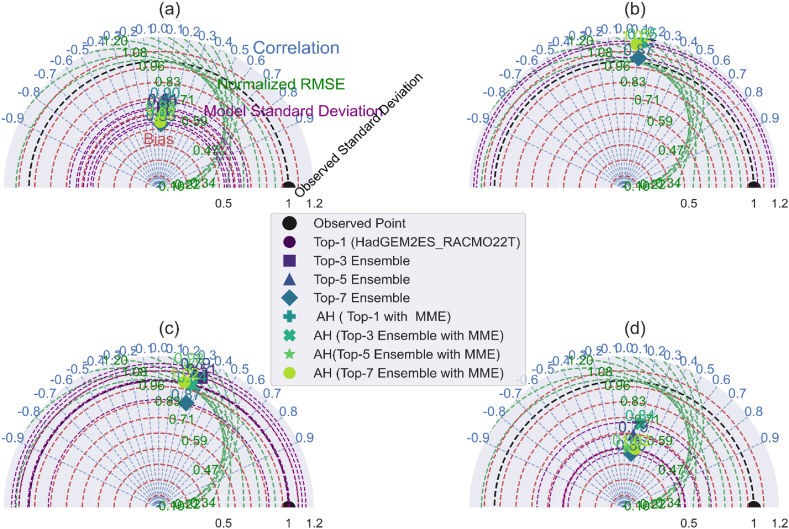
Taylor Diagram: AFR44 RCMs performance evaluation for minimum temperature over 1991–2005: (a). Daily (b). Monthly (c). Seasonally (d). Yearly.

**Fig. 9:**
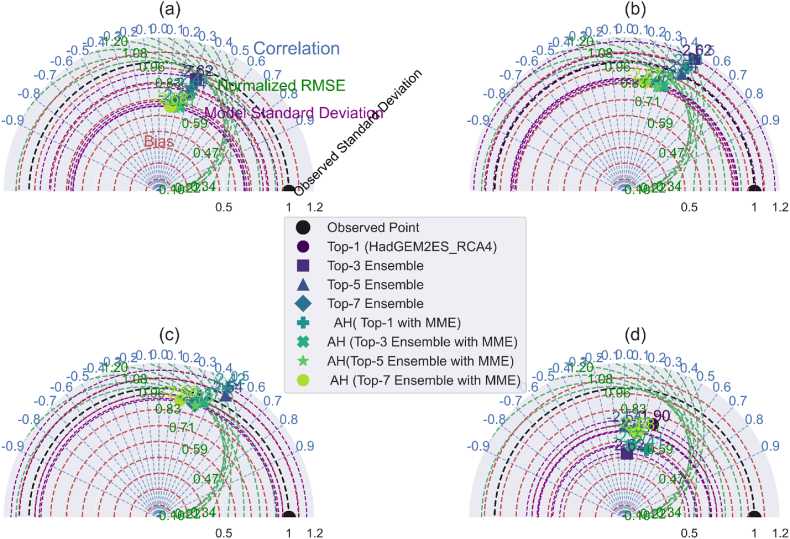
Taylor Diagram: AFR44 RCMs performance evaluation for maximum temperature over 1991–2005: (a). Daily (b). Monthly (c). Seasonally (d). Yearly.

**Fig. 10:**
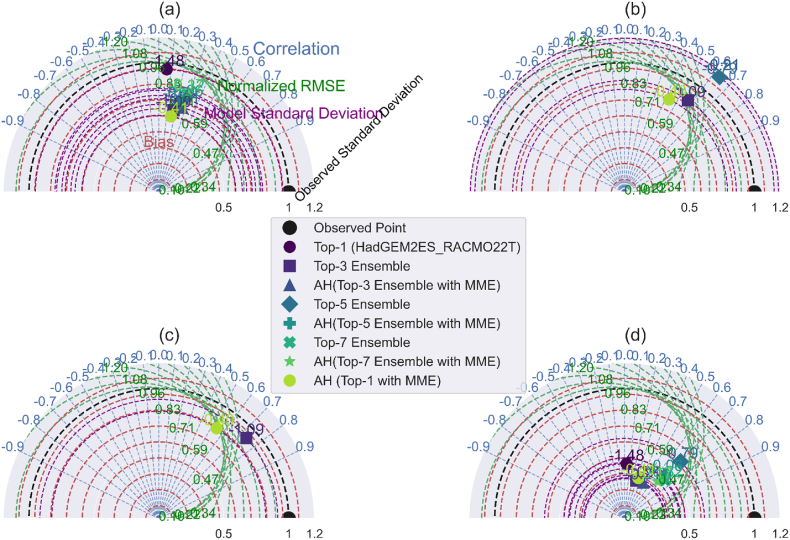
Taylor Diagram: AFR44 RCMs performance evaluation for precipitation (1991–2005) (a). Daily (b). Monthly (c). Seasonally (d). Yearly.

Taylor diagram through the 2D plot using the correlation coefficient, centered root-mean-square error, and model-observed standard deviation ratio. Fig. 8(a–d), Fig. 9(a–d), and Fig. 10(a–d) illustrate the comparison of minimum temperature, maximum temperature, and precipitation using the Taylor diagrams plot for the AFR-44 RCMs. Basically, best-performing approach or model standard deviation is close to reference data, their low-centered root means square difference, and their correlation coefficient is high. Through this the ensemble creation of model combinations among these approaches informs better model selection than a single model or MME.

Taylor diagrams are powerful multidimensional evaluation tools that demonstrate the performance of the best model predictions with observed data using the Pearson correlation coefficient, root-mean-square error (RMSE), and standard deviation. In the context of precipitation, the AH (top-1 with MME) strategy stands out for its strong correlation with observed daily values, as well as its low RMSE and attractive standard deviation ratio (RSR). This combination demonstrates the model's dependability in predicting daily variability, which well aligns with observed precipitation patterns.

In contrast, the top-3 ensemble exhibits its strength at the monthly temporal scale, with a good correlation, low RMSE, and competitive RSR. These characteristics demonstrate its durability in simulating average monthly trends while remaining consistent with observed data. For seasonal and yearly precipitation forecasts, the top-1 (HadGEM2ES_RACMO22T) stands out as the best model, successfully reflecting observed patterns with high correlation, low RMSE, and an excellent RSR, demonstrating its ability to represent both seasonal fluctuations and annual totals.

In terms of maximum temperature, the top-7 ensemble performs admirably, with strong correlation and variance for both monthly and seasonal predictions, as well as a low RMSE and good RSR. This strengthens its ability to capture temperature extremes across these temporal spans. Meanwhile, the top-1 (HadGEM2ES_RACMO22T) stands out on an annual scale, with a good correlation, low RMSE, and a remarkable RSR, proving its dependability in forecasting yearly temperature trends. The top-3 ensemble also performs well in forecasting lowest temperature across various temporal scales, with good correlation and low RMSE, confirming its standing as the best-performing technique in this situation.

## 4 Discussions

Overall, a multi-metric ranking used to measure the efficiency of the CORDEX-AFR44 models reveals inconsistency when relying solely on a single climate model. This implies that there is inherent uncertainty in climate projections, which can be handled by employing numerous GCMs and RCMs. Because of the dynamic nature of climate change, averaging datasets from climate models is critical for long-term assessment. Methods such as multi-metric weighted ranking, empirical cumulative distribution functions (empirical CDF), Taylor skill scores, and Taylor diagrams aid in selecting appropriate RCM simulations from AFR-44 for evaluating climate models and reference data. As a result, techniques such as average hybrid and specific multi-model ensembles (SMME) are preferred for simulating climatic variables on a short time scale (daily, monthly, and seasonal), with a single model outperforming long-term (yearly) forecast.

This study's findings are important for future research into climate model assessments and their implications in southeast Ethiopia. Combining particular models based on average hybrid MME with top-N ensemble and SMME (top-N ensemble) techniques is appropriate for selecting RCMs for the area of interest. These ensemble approaches are advised to reduce uncertainty in climate models.

For paired GCM-RCM combinations employing hybrid (average prediction MME with top-N ensemble) and SMME (top-N ensemble) techniques, RCA4 and RACMO22T are effective Regional Climate Models (RCMs) in simulating air temperatures and precipitation in the Melka-Wakena Catchment in Southeast Ethiopia. Common driving GCMs and their categories provide insights into their suitability for precipitation, maximum, and minimum temperatures. HadGEM2ES, IPSLCM5AMR, EARTH, and CNRMCM5 is a key driving GCM for all three variables and falls under RCA4 and RACMO22T**,** supporting its robustness as highlighted by previous studies and identified these models and emphasized their strength in regional [2,7,32,37,[45], [46], [47]].

This study provides valuable regional implications. Understanding the performance of these appropriate driving GCMs and RCMs enhances projections for water resource management, agriculture, and other climate-sensitive sectors. Accurate climate modeling can improve strategies for managing water resources, optimizing agricultural practices, and preparing for climate variability, thereby supporting more effective adaptation and mitigation efforts in the region.

## 5 Conclusion

• Climate science helps support policy and decision-making, adaptation issues, mitigation planning and risk management strategies. Climate model uncertainty occurs at the Melka-Wakena Catchment in southeast Ethiopia. In this study, EMA, and ESGF provide the observed and climate model datasets for 1991–2005.
• Performance indicators such as evaluation multi-metrics, empirical CDF, Taylor skill score, and Taylor diagrams were helpful in evaluating the CORDEX Africa regional climate model.
• The research emphasizes that the best model for rainfall is not the same as the best model for minimum and maximum temperature for paired GCM-RCM. Also, the finding shows that all performance indicators using the evaluation metrics are inconsistent over a single model. For each climate variable, the best individual model, multi-model ensemble, specific multi-model ensemble (top- N ensemble) and averages hybrid approaches are useful for evaluating the performance of RCM simulation from CORDEX-Africa.
• In addition, Taylor diagram and Taylor Skill Score (TSS) was providing a comprehensive indicator of model performance, and resulting ensemble methods generally offer robustness and accuracy, single-model approaches can be highly effective for specific temporal periods in the simulation of climatic conditions and helpful in reducing climate model uncertainty.
• Further study of, impacts, adaptations and mitigation of climate change may be possible using this dataset for Melka-Wakena catchment south east Ethiopia.
• Based on the ideas outlined above, we present the subset of GCM-RCMs most relevant to the Africa CORDEX project in its south-east Ethiopia study areas, as well as describe the selection procedure used to identify the possible model selection.

## CRediT authorship contribution statement

**Tadele Sh Gerasu:** Writing – original draft, Visualization, Validation, Software, Methodology, Investigation, Formal analysis, Data curation. **Tolera Abdissa Feyissa:** Writing – review & editing, Conceptualization. **Beekan Gurmessa Gudeta:** Writing – review & editing, Conceptualization. **Keneni Demissie:** Data curation. **Mulatu Tesfahun:** Methodology.

## Data availability statement

The observed data used in this study are available upon request from the corresponding author via email at t4shgeresu@gmail.com. CORDEX Africa domain data can be accessed publicly through the Earth System Grid Federation (ESGF) at https://esgf-node.llnl.gov. Furthermore, the research data associated with this study has been deposited in a publicly available repository: Zenodo, and can be accessed via the following URL: https://doi.org/10.5281/zenodo.14208274 [48]. Researchers are encouraged to use these datasets for further investigations to validate and extend the findings of this study.

## Ethical statement

The submitted article is our original work and has not been published previously, and that it is not under consideration for publication elsewhere. Any supported resource used for this work are cited relevant previously published work appropriately. We checked for ethical approval for this study because "the research conducted in our institutions does not violate ethics." Also, this research did not involve the use of animal experimentation or human subjects.

## Funding

"This research was funded by Jimma University, Jimma Institute of Technology, and the Research Publication Director Office."

## Declaration of competing interest

The author(s) declare no competing interests.
